# Parkinson-ALS with a novel MAPT variant

**DOI:** 10.1007/s10072-023-07081-4

**Published:** 2023-09-20

**Authors:** Camilla Ferrari, Assunta Ingannato, Sabrina Matà, Silvia Ramat, Luca Caremani, Silvia Bagnoli, Valentina Bessi, Sandro Sorbi, Benedetta Nacmias

**Affiliations:** 1 Department of Neuroscience, Psychology, Drug Research and Child Health, University of Florence, 50134 Florence, Italy; 2 Neuromuscular-Skeletal and Sensory Organs Department, AOU Careggi, Florence, Italy; 3 Parkinson Unit, Neuromuscular-Skeletal and Sensory Organs Department, AOU Careggi, Florence, Italy; 4 IRCCS Fondazione Don Carlo Gnocchi, 50143 Florence, Italy

**Keywords:** MAPT, PD-ALS, Brait–Fahn–Schwarz disease, Clinical complex

## Abstract

The mutations on microtubule associated protein tau (*MAPT*) gene manifest clinically with behavioural frontotemporal dementia (FTD), parkinsonism, such as progressive supranuclear palsy and corticobasal degeneration, and rarely with amyotrophic lateral sclerosis (ALS). FTD-parkinsonism and FTD-ALS are clinical overlaps included in the spectrum of *MAPT* mutation’s phenotypes. The mutations on *MAPT* gene cause the dysfunction of tau protein determining its accumulation in neurofibrillary tangles. Recent data describe frequently the co-occurrence of the aggregation of tau protein and α-synuclein in patients with parkinsonism and Parkinson disease (PD), suggesting an interaction of the two proteins in determining neurodegenerative process. The sporadic description of PD-ALS clinical complex, known as Brait–Fahn–Schwarz disease, supports the hypothesis of common neuropathological pathways between different disorders. Here we report the case of a 54-year-old Italian woman with idiopathic PD later complicated by ALS carrying a novel *MAPT* variant (Pro494Leu). The variant is characterized by an amino acid substitution and is classified as damaging for MAPT functions. The case supports the hypothesis of tau dysfunction as the basis of multiple neurodegenerative disorders.

## Introduction

The overlap of clinical syndromes is not rare, especially among neurodegenerative disorders that share neuropathological features and genetic determinants. Frontotemporal dementia (FTD)-parkinsonism and amyotrophic lateral sclerosis (ALS)-FTD are typical examples [1, 2]. Instead, the association between Parkinson disease (PD) and ALS, a complex termed Brait–Fahn–Schwarz disease (BFS) is extremely rare [3–8]. After the first description [3], few cases have been sporadically reported worldwide [4–8]. Neuropathological information on BFS patients is scarce and controversial [3–8], and a genetic link has not been identified so far.

Here, we report the first case of a 54-year-old Italian woman affected by BFS disease (PD-ALS) carrying a novel variant of the microtubule associated protein tau (*MAPT)* gene.

## Materials and methods

Genetics: Genomic DNA was isolated from peripheral blood sample using a standard automatic method (QIAcube, QIAGEN). Initially, *MAPT* gene and progranulin (*GRN*) gene were PCR amplified and analysed by Sanger sequencing on SeqStudio Genetic Analyzer (Thermo Fisher, Waltham, MA, USA). Subsequently, a more thorough sequencing of the patient DNA was performed with a custom next-generation sequencing (NGS) panel of 73 dementia-related genes. Sample library was prepared using the Illumina® DNA Prep with Enrichment kit. Reads were aligned to GRCh37/hg19 reference genome. Variants were annotated with ANNOVAR [9], and allele frequency was checked on population databases (1000Genomes, ESP, and gnomAD) [10]. In silico prediction tools (SIFT, PolyPhen-2, REVEL, Mutation Assessor, CADD, and MetaLR) were used to assess non-synonymous variants and their possible effect on protein structure and function. Moreover, the apolipoprotein E (*APOE*) haplotype was investigated by high-resolution melting analysis (HRMA). The regions encompassing rs7412 [NC_000019.9:g.45412079C>T] and rs429358 (NC_000019.9:g.45411941T>C) of APOE were PCR amplified using two sets of designed primers. Samples with known APOE haplotype were used as standard references.

## Case report

### Clinical features

**Fig. 1:**
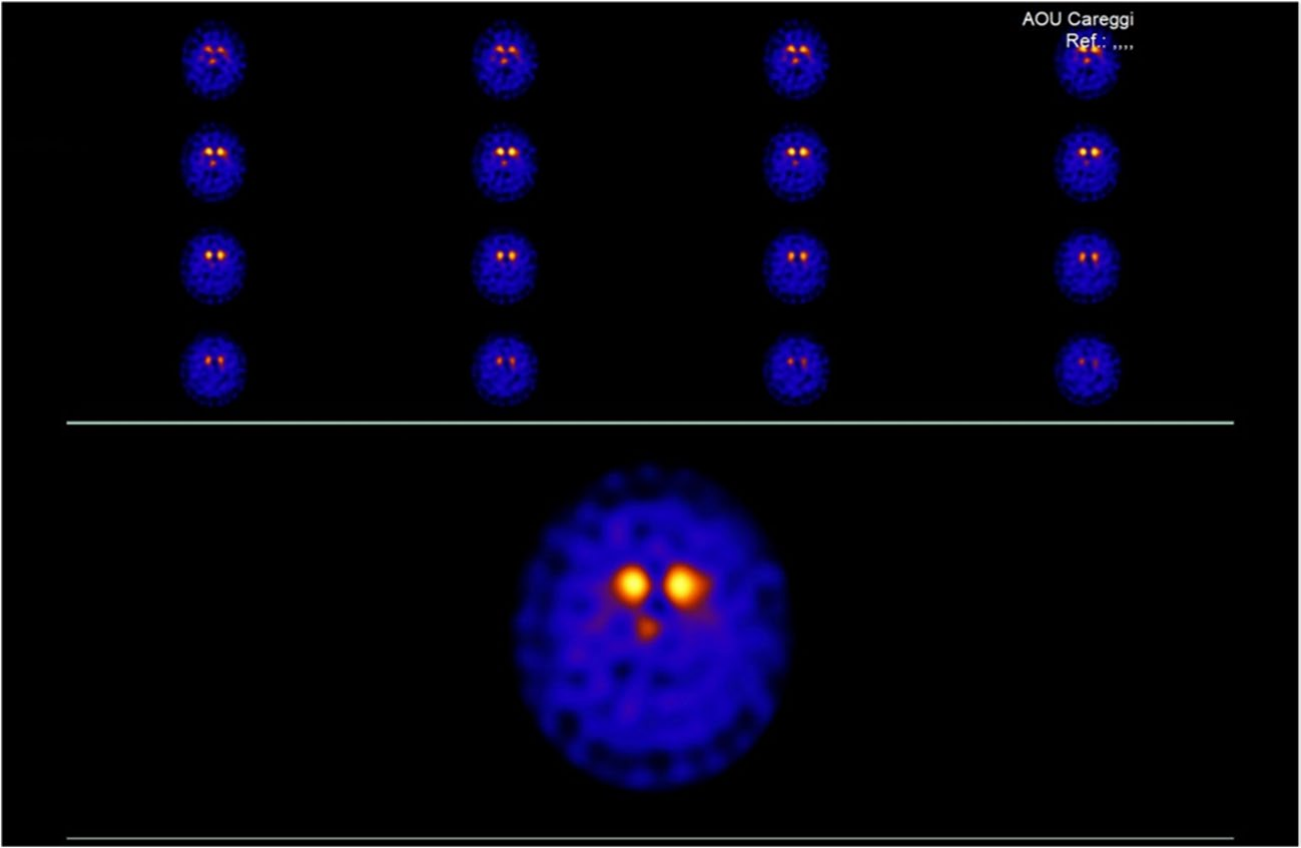
The dopamine transporter brain imaging with single-photon emission computed tomography (DAT-SPECT) revealing reduced uptake in both putamen nuclei, mostly in right putamen. Volume distribution (VD): right caudate 2.33, left caudate 2.35, right putamen 0.71, left putamen 0.88 (normal VD values: right caudate 2.58±0.41; left caudate 2.57±0.37; right putamen 2.14±0.41; left putamen 2.23±0.45)

A 54-year-old healthy woman developed resting tremor of the left hand, then tremor of the left leg, and slight bradykinesia of the left limbs. Brain magnetic resonance imaging (MRI) detected mild microvascular ischemic changes of the white matter, while the dopamine transporter brain imaging with single-photon emission computed tomography (DAT-SPECT) revealed reduced uptake in both putamen nuclei, mostly in right putamen (Fig. 1). The 123I-metaiodobenzylguanidine (MIBG) myocardial scintigraphy showed an impairment of the cardiac sympathetic nerve fibres. Electromyography (EMG) examination and motor evoked potentials (MEP) were normal, as well as the cognitive profile. The patient was treated with rasagiline 1 mg a day and long-lasting pramipexole up to 2.1 mg a day. The treatment was effective and well tolerated. Based on PD diagnostic criteria [11], a diagnosis of idiopathic PD was made. After 2 years of clinical stability, the patient complained frequent falls due to weakness of lower limbs. Gait became rapidly difficult, and at the age of 58 years, she could stand up only with help and could perform only few steps. Six months later, she was bedridden due to progressive weakness of the four limbs. Tendon reflexes were increased in upper and lower limbs, and Hoffmann and Babinski signs were bilaterally present. Increased muscle tone, with both spasticity and rigidity, was detected in the four limbs, with prevalence on the left side. Muscular atrophy and fasciculations were observed on both deltoids and quadriceps muscles. Her speech was dysarthric and hypophonic. Brain MRI resulted unchanged and cervical MRI ruled out spinal lesions. Total body CT scan with contrast medium did not detect any abnormalities in the brain, neck, chest, and abdomen. Liver, kidney, renal, and thyroid functions were normal. An infective assessment was performed in the blood (HCV, HBV, syphilis, *Borrelia*, HIV, HTLV) and in the cerebrospinal fluid (syphilis, HSV, EBV, VZV, *Borrelia*). No infections were detected. The screening for autoantibody included anti-Hu, anti-Yo, anti-Ri, anti-CV2, anti-Anfifisina, anti-NMDA, anti-LG1, anti-CASPR, anti-GABA, anti-GluR1, anti-GluR2, and anti-gangliosides (GQ1b, GT1b, GT1a, GD3, GD1b, GD1a, GM3, GM2, GM1) and gave negative results. Electrodiagnostic studies showed reduction of compound muscle action potential (CMAP) amplitudes in most motor nerves examined (right and left tibial, peroneus, median, and ulnar nerves) from 4 limbs, with normal conduction velocity. Sensory nerve conduction study was normal. Standard needle EMG examination, performed in the bulbar and thoracic regions, and at proximal and distal muscles from each limb, showed diffuse fibrillation and fasciculation potentials in the upper and lower extremities, associated to chronic motor unit changes. MEPs, measured from posterior tibial and ulnar nerves, resulted of reduced amplitude as compared to distal CMAP values, with increased central conduction time. A diagnosis of definite ALS was made according to revised El Escorial criteria [12]. Cognitive evaluation was not performed due to the severe dysarthria; however, the patient was oriented in time, space and toward people, but apathy, loss of empathy, and inappropriate laughing and crying were detected. Cerebrospinal fluid was normal including neurodegenerative biomarkers of amyloid and tau protein: Aβ_42_ 660 pg/ml (n.v >670 pg/ml); Aβ_42_/Aβ_40_ 0.10 (n.v > 0.062) ; total-tau 247 pg/ml (n.v < 400 pg/ml); phosphorylated-tau 181 13.3 pg/ml (n.v.< 60 pg/ml) [13]. A conclusive diagnosis of ALS associated with PD was made. To complete the diagnostic process, genetic analysis with a panel of 73 dementia-related genes was performed, including genes associated with FTD and ALS (*GRN, C9orf72, SOD1, TARDBP, MAPT*). After the discharge from the hospital, the clinical condition of the patient rapidly deteriorated, and swallowing and breathing difficulties required the insertion of a gastric tube and the use of non-invasive ventilation. Ten months later, at the age of 59 years, she died of respiratory failure. A request for autopsy was declined by family members.

### Genetic features

The patient was found to carry a novel missense variant in exon 9 of *MAPT* gene, NM_016835.4:c.1481C>T, p.(Pro494Leu). The p.(Pro494Leu), rs763728305 indbSNP, is classified as variant with an uncertain significance according to the American College of Medical Genetics and Genomics guidelines. SIFT and PolyPhen-2 predict that the variant is deleterious/possible damaging for protein structure and function (SIFT score= 0.01, scores <0.05 are predicted to influence protein activity; PolyPhen-2 score= 0.465, score ranges from 0.0=tolerated to 1.0=deleterious), instead REVEL, Mutation Assessor, and MetaLR evaluate the variant as likely benign/tolerated. The residue is conserved (phyloP100way = 6.033, greater than 3.81 cutoff). CADD v1.6 indicate a 22 score; C-score≥ 20 indicates the 1% most deleterious substitution in human genome. The variant p.(Pro494Leu) was not reported in ClinVar database and has a frequency in general population of ƒ = 0.00000398 (GnomAD). Sequencing not evidenced other significant variants in the patient, APOE haplotype resulted ε3/ε3. Family history was negative for neurological disorders: the father died at 94 years, while the mother died at young age at 54 years of haematological neoplasm. The patient’s relatives, a healthy 5-year-old brother and two daughters, preferred not to undergo the genetic testing.

## Discussion

We presented the case of 54-year-old Italian woman with levodopa-responsive parkinsonism who later developed clinical symptoms consistent with ALS. The initial clinical suspicion of idiopathic PD was supported by the responsiveness to levodopa and the impairment of sympathetic innervation at myocardial scintigraphy, a method used to differentiate between atypical parkinsonism and PD [14]. Moreover, the phenotype of MND fitted the revised El Escorial criteria for ALS [12]. The clinical picture resembles the phenotype of the BFS disease [3] characterized by PD followed after 1–2 years by ALS. The BFS disease has been described in few case reports [3, 5, 6, 8] and screened in two cohort studies [4, 7], for a total of no more than 50 cases since its first description in 1973 [3]. In the first cohort study, 27 of 5.500 patients with parkinsonism also had MND, only seven of whom had PD-ALS complex [4]. In the other cohort study, among 1042 patients diagnosed with ALS or other MNDs, six had concomitant diagnosis of PD [7].

No evidence of mutations in genes associated with FTD-ALS (*SOD1, FUS, angiogenin, GRN*) nor with PD (*Park-1, Park-2, Park-6, Park-7, DJ-1*) were detected so far. *MAPT* gene was not screened in the total of the cases [5, 6, 8]. Pasquini et al. reported one mutation in *C9orf72* gene and one in *TARDP* gene, but it is not specified if they refer to patients with atypical parkinsonism-MND or with PD-ALS [7]. Our patient was found to carry a novel variant of *MAPT* gene causing an amino acid substitution in exon 9 with a potential pathological role, as predicted by the in silico prediction tools.

The *MAPT* gene encodes for protein Tau and exon 9 is placed in the microtubule (MT)-binding domain. Mutations in the MT-binding domain resulted in microtubules destabilization, unbound tau, and consequently tau aggregation [15]. Neurodegenerative disorders associated with *MAPT* mutations are bvFTD, primary progressive aphasia, progressive supranuclear palsy, corticobasal degeneration, and the FTD-parkinsonism and FTD-ALS clinical complexes [1, 2]. All are characterized by the accumulation of hyperphosphorylated tau (neurofibrillary tangles, NFTs). The role of *MAPT* in pure ALS is less characterized than in the FTD-ALS complex; however, recently, a *MAPT* mutation was identified in two unrelated ALS Italian patients [16, 17]. *MAPT* mutations have never been described in patients with PD, but PD patients have been found to present both α-synuclein and NFTs aggregations [2]. Interestingly, Tau protein, behind its functions related to microtubules, is involved in common hallmarks of neurodegeneration processes, such as increased oxidative stress and mitochondrial dysfunction, suggesting a crucial role of tau in the pathogenesis of different neurodegenerative disorders. In vivo and in vitro data reported that the dysfunction of tau could produce different kinds of proteinopathies [2, 15, 18] The lack of neuropathological data does not allow us to confirm this hypothesis. Moreover, the pathogenicity of this novel *MAPT* variant is uncertain, as we don’t have information on the genetic status of family members and RNA sample of the patient for molecular analysis was not available.

However, if this variant is not the cause of this clinical complex, we think it can still represent a risk factor. Previous studies have already reported that *MAPT* haplotype and *MAPT* variants influence the risk of both PD [2, 18, 19] and ALS [18, 20–22]. Even if a *MAPT* variant is silent at protein level, it can affect the level of tau and the post-transcriptional modifications and can influence the interaction between tau and other proteins [18]. The level of tau and its phosphorylation are supposed to influence the interaction between tau and α-synuclein and their synergistic fibrillization [2, 18, 23].

This is the first case in which a variant of the *MAPT* gene (Pro494Leu) was associated with a BFS’s phenotype, idiopathic PD followed by ALS. This variant of *MAPT* gene has never been identified before, and if not, the cause of the syndrome could be, at least, a risk factor, supporting the crucial role of tau in the development of different neurodegenerative disorders.

## Data Availability

The data present in this article are available on reasoned request from the corresponding author.
